# Association of low vitamin D level and full-term early-onset neonatal sepsis; a case-control study

**DOI:** 10.1186/s13052-024-01665-2

**Published:** 2024-05-18

**Authors:** Shereen A. Mohamed, Nermin R. Kamel, Aya E. Fouda, Rabab E. Elhawary, Mohamed A. Abdelmegeid

**Affiliations:** 1 Pediatrics Department, Kasr Alainy Faculty of Medicine, Cairo University, Cairo, Egypt; 2 Clinical Pathology Department, Kasr Alainy Faculty of Medicine, Cairo University, Cairo, Egypt

**Keywords:** Mortality, Vitamin D deficiency, Neonatal Sepsis

## Abstract

**Background:**

Sepsis is one of the main causes of death in newborns worldwide. Vitamin D levels during fetal and neonatal periods have a significant role in the development of the immunological system. The study aims to evaluate the association between vitamin D levels and the risk of early-onset neonatal sepsis in full-term neonates in a developing country.

**Methods:**

This case–control study was conducted at the Neonatal Intensive Care Units (NICUs) of Kasr Alainy Hospital, Cairo, Egypt. The study was composed of two groups; the sepsis group involved full-term neonates appropriate for gestational age with sepsis-related clinical signs. The control group included newborns with no signs of clinical/laboratory infection within 72 h of life. Blood samples were collected on admission during the first three days of life in both groups for the measurement of 25-hydroxyvitamin D levels, Complete Blood Count (CBC), C reactive protein (CRP), and blood culture.

**Results:**

Forty-five newborns with clinical and laboratory findings of early-onset neonatal sepsis within 72 h of life were enrolled, and the control group included forty-five newborns with no evidence of sepsis. Vitamin D levels in the sepsis group were significantly lower than in the control group. Apgar score at the first minute was significantly lower in the sepsis group. 57.8% of neonates with sepsis had positive blood cultures. There was a statistical difference between deficient, insufficient, and sufficient vitamin D levels regarding the duration of the NICU stay, which was longer in neonates with deficient vitamin D levels. CRP was significantly higher in neonates with deficient vitamin D levels. The area under the receiver operating characteristic curve for serum vitamin D in the prediction of neonatal sepsis was 0.76 at a cutoff < 19.7(ng/ml).

**Conclusion:**

In the current study, full-term newborns with EOS had considerably lower vitamin D levels than healthy controls. Through appropriate vitamin supplementation of the mothers during pregnancy, it could be possible to ensure adequate vitamin D levels for newborns. This may contribute to the reduction of the risk of EOS, together with the other well-known preventive measures (i.e. breastfeeding and intrapartum antibiotic prophylaxis).

## Background

Neonatal sepsis is a clinical term describing non-specific symptoms and signs of infection that may be proven with a positive culture [1]. The early-onset sepsis (EOS) occurs in the first three days of life [1]. Low and middle-income countries have high mortality rates among neonates with sepsis [2]. Also in high-income countries infections and sepsis represent a relevant cause of mortality and morbidity, especially in those peripheral and rural areas with low-density offers of second-level perinatal care [3].

Immature immunological systems predispose newborn infants to sepsis. The hypothesis relating vitamin D deficiency with the raised incidence of infections was assumed for over a century. Its levels during the prenatal and neonatal periods have been associated with the development of the immune system. Vitamin D levels may have an impact on the production of immunoglobulins, T cell lymphocyte activation, killer and T helper cell differentiation, neutrophil migration, and the production of antimicrobial peptides. Moreover, cord-blood 25-hydroxyvitamin D low levels have been related to the risk of early-onset neonatal sepsis [4–6].

Several studies were carried out to determine the relationship between low vitamin D concentrations and the likelihood of early-onset neonatal sepsis and sepsis indicators [5, 7–11].

This study aims to analyze the association between different vitamin D levels and increased risk of early-onset neonatal sepsis in full-term neonates appropriate for gestational age (AGA) in Egypt.

## Methods

This case–control study was conducted at Neonatal Intensive Care Units (NICUs) of Kasr ElAiny Hospital, Cairo University. All participants were enrolled within one year.

The study included full-term AGA neonates. The enrolled population was divided into two groups; the sepsis group included full-term AGA neonates with sepsis-related clinical signs; temperature instability, tachycardia or bradycardia, hypotension, need for supplemented oxygen, apnea, need for mechanical ventilation, abdominal distension, feeding intolerance, necrotizing enterocolitis, serum parameters other than C reactive protein (CRP): white blood cell count, absolute neutrophil count, platelets count (Table 1 shows the clinical and laboratory criteria for EOS) [12]. The control group included newborns showing no signs of infection or laboratory markers of infection within 72 h of birth.

**Table 1:** Clinical and laboratory findings of EOS [12]

Groups	Criteria
**Highly probable sepsis**	At least three clinical signs related to sepsis
	CRP > 1 mg/dl
	At least another two altered serum parameters + CRP
	Positive or negative blood culture
**Probable sepsis**	Less than 3 clinical signs related to sepsis
	CRP > 1 mg/dl
	At least another two altered serum parameters + CRP
	Negative blood culture
**Possible sepsis**	Less than 3 clinical signs related to sepsis
	CRP < 1 mg dl
	Less than 2 others altered serum parameters
	Negative blood culture
**No sepsis**	No clinical signs related to sepsis
	CRP < 1 mg/dl
	No altered serum parameters
	Negative blood culture

Clinical signs related to sepsis; temperature instability, tachycardia or bradycardia, hypotension, need for supplemented oxygen, apnea, need for mechanical ventilation, abdominal distension, feeding intolerance, necrotizing enterocolitis, serum parameters other than C- C-reactive protein (CRP): white blood cell count, absolute neutrophil count, platelets count

Exclusion criteria included neonates admitted after 72 h of life, newborns with major congenital anomalies for possible renal and hepatic involvement that might influence vitamin D levels, and genetic diseases especially those affecting the immunological system [13]. Maternal and perinatal factors (urine tract infection, chorioamnionitis, premature rupture of membrane, meconium-stained amniotic fluid, prolonged labor, and neonates with small gestational age) were also excluded for increased risks of adverse effects, including neonatal sepsis [14].

For all participants, careful history was recorded including perinatal data (gestational age, mode of delivery, resuscitation maneuvers, and Apgar score), and postnatal ones (activity, suckling, and NICU admission).

A thorough clinical examination was necessary to find the clinical signs of sepsis, which included agitation, lethargy, hypotonia, pallor or mottled skin, hypothermia or hyperthermia, apnea or tachypnea, tachycardia or bradycardia, poor oral intake, jaundice, and intestinal obstruction [15]. Newborns were followed during their NICU stay period.

The following laboratory investigations were performed: CRP, blood culture, and complete blood count (CBC). Newborns fulfilling the inclusion criteria and admitted during their first three postnatal days of life in both groups at admission underwent blood sample collection and a level of 25_hydroxyvitamin D (25-OHD) test. After centrifugation, the serum was separated, and it was kept at -20 °C until testing. ELISA kit** (**DiaMetra DKO146) was utilized according to the manufacturer. The vitamin D concentration in the sample was calculated through a calibration curve.

Vitamin D level was considered sufficient if it was more than 30 ng/ml, insufficient level laid between 20 to 30 ng/ml, and deficient level if less than 20 ng/ml.

### Statistical analysis

The IBM SPSS (Statistical Package for Social Science) version 25 for Windows was used to code, compute, and analyze the data. Frequency tables (numbers and percentages) were used to display descriptive statistics for qualitative data. The Shapiro–Wilk test was used to determine the normality of the data for the quantitative variables, and the results were displayed as standard deviations (SD). The Median (minimum–maximum) was used for variables with non-normal distributions.

Chi-square test was used in analytical statistics to assess the relationship between categorical variables. If the Excel cell count in four-cell tables was less than 5, the Fisher Exact Test has been used instead. The Monte Carlo test was used if the expected cell count in more than four-cell tables was less than 5.

An independent sample t-test was used in two independent groups to investigate the association between normally distributed continuous variables. Two independent continuous variables that were not normally distributed were compared using the Mann–Whitney U test (z). Non-normally distributed data were correlated using the Spearman correlation method.

To ascertain whether there were any statistically significant differences between the means of two or more independent (unrelated) groups, the one-way analysis of variance (ANOVA) was utilized. Additionally, the Kruskal–Wallis H Test was employed to compare continuous variables with non-normal distribution across many groups.

To compute the sensitivity and specificity rates and, consequently, the positive and negative probability ratios of such variables, receiver operating characteristic (ROC) curves were used to evaluate the overall prediction accuracy of test parameters, as demonstrated by the area under the curve (AUC).

The prediction of sepsis' independent factors was carried out using binary stepwise logistic regression analysis. The forward Wald approach was used to add significant predictors from the univariate analysis to the regression model.

Generated odds ratios with a 95% confidence interval were adjusted odds ratios (AOR).

Results were deemed significant for all the statistical tests when the chance of error was less than or equal to 5% (*p* ≤ 0.05).

## Results

This case–control study was conducted at Neonatal Intensive Care Units (NICUs) of Kasr Alainy Hospital of Cairo University, Egypt. Ninety newborns were enrolled and divided into two groups. The sepsis group included forty-five newborns with clinical and laboratory findings of EOS within 72 h of life. The control group included forty-five newborns with no signs of clinical-laboratory signs of infection within the same time window.

There was no statistically significant difference between the two groups regarding gestational age, gender, consanguinity, mode of delivery, Apgar score at 5 min, temperature, and hemoglobin level. However, there was a significant difference between the two groups regarding Apgar score at the first minute, total leucocytic count (significantly higher within the sepsis group), and platelet count (significantly lower within the newborns with sepsis)**.** Jaundice was the most prevalent clinical manifestation in the control group (88.9%), whereas respiratory distress predominated in the sepsis group (64.4%). CRP was increased within the sepsis group. Serum vitamin D levels were significantly lower in the cases with sepsis compared to the control ones (*p* < 0.001) (Table 2).

**Table 2:** Features, clinical and laboratory data of the studied group (*N* = 90)

	**Sepsis Group** ***N*** ** = 45**	**Control group** ***N*** ** = 45**	**Test of significance**
**Gestational age ** ***(weeks)***			
***Mean*** ** ± ** ***SD***	**37.5 ± 0.55**	**37.5 ± 0.59**	**t = 0.37**
*** (Min–max)***	**(37–39)**	**(37–39)**	***P*** ** = 0.71**
**Gender**			
**• Male**	**19(42.2%)**	**23 (51.1%)**	**χ**^**2**^** = 0.71**
**• Female**	**26(57.8%)**	**22 (48.9%)**	***P*** ** = 0.39**
**Consanguinity**			
• **Negative**	40 (88.9%)	35 (77.8%)	χ^2^ = 2.0
• **Positive**	5 (11.1%)	10 (22.2%)	*P* = 0.16
**Mode of delivery**			
• **Normal vaginal delivery**	4 (8.9%)	6 (13.3%)	χ^2^ = 0.45
• **Cesarean section**	41 (91.1%)	39 (86.7%)	*P* = 0.50
**Apgar score (at 1 min)**			
mean ± SD	8.0 ± 0.88	8.5 ± 0.73	t = 2.6
(Min–max)	(5–9)	(7, 8, 9)	***P*** = 0.01*
**Apgar score (at 5 min)**			
mean ± SD	9.4 ± 0.58	9.6 ± 0.49	t = 1.9
(Min–max)	(8, 9, 10)	(9, 10)	*P* = 0.053
**Temperature**(^o^C)			
mean ± SD	37.1 ± 0.21	37 ± 0	t = 1.4
(Min–max)	(37, 38)	(37–37)	*P* = 0.16
**Hemoglobin level**(g/dL)			
mean ± SD	13.1 ± 2.1	13.2 ± 1.4	t = 0.21
(Min–max)	(6–18.7)	(10.2–16)	*P* = 0.84
**Total leucocytic count**(10^3^/mm^3^)			
mean ± SD	18.2 ± 5.4	12.0 ± 1.9	t = 7.1
(Min–max)	(2.7–29.4)	(6.9–15.9)	***P*** ≤ 0.001*
**Platelet count**(10^3^/mm^3^)			
mean ± SD	185.9 ± 55.5	356.3 ± 55.1	t = 11.2
(Min–max)	(77–420)	(155–459)	***P*** ≤ 0.001*
**Primary clinical manifestations**			
• **Respiratory distress**	29 (64.4%)	0	MC = 51.7
• **Jaundice**	9 (20%)	40 (88.9%)	***P*** ≤ 0.001*
• **Symptoms of sepsis**	3(6.7%)	0	
• **Transient tachypnea of the newborn**	4(8.9%)	5 (11.1%)	
**Poor feeding**	30 (66.7%)	4 (8.9%)	χ^2^ = 31.9 ***P*** ≤ 0.001*
**Respiratory difficulties**	34 (75.6%)	5 (11.1%)	χ^2^ = 38.1 ***P*** ≤ 0.001*
**Jaundice**	12 (26.7%)	44 (97.8%)	FET ***P*** ≤ 0.001*
**Lethargy**	30 (66.7%)	2 (4.4%)	FET ***P*** ≤ 0.001*
**Cardiovascular symptoms of sepsis** **(**tachycardia or bradycardia & hypotension)	34 (75.6%)	3 (6.7%)	χ^2^ = 44.1 ***P*** ≤ 0.001*
**Mottling**	35 (77.8%)	1 (2.2%)	FET ***P*** ≤ 0.001*
**CRP Positive**	45 (100%)	0	FET ***P*** ≤ 0.001*
**CRP level in sepsis group**(g/dl) Median (min–max)	24 (6–96)		
**Serum Vitamin D level**(ng/ml) Median (Min – max)	15.4 (6.1–57.2)	30.4 (5.1–107)	Z = 4.2 ***P*** ≤ 0.001*
**Vitamin D status**			
• **Deficient level (< 20** ng/ml**)**	30 (66.7%)	7 (15.6%)	χ^2^ = 24.3
• **Insufficient level (20–30** ng/ml**)**	5 (11.1%)	14 (31.1%)	***P*** ≤ 0.001*
• **Sufficient level (> 30** ng/ml**)**	10 (22.2%)	24 (53.3%)	
**Length of NICU stay**(days) Median (Min–max)	13 (8, 9, 10, 11, 12, 13, 14, 15, 16, 17, 18, 19, 20, 21, 22)	6 (2, 3, 4, 5, 6, 7, 8, 9, 10)	Z = 7.8 ***P*** ≤ 0.001*

Twenty-six (57.8%) neonates with sepsis had positive blood cultures, and 19(42.2%) had no growth results (Table 3).

**Table 3:** Blood culture results of the sepsis group (*N* = 45)

	**Items**	*N* (%)
**Blood culture**	• No growth	19 (42.2%)
	• Positive	26 (57.8%)
**Type of organism**	• No growth	19 (42.2%)
	• Klebsiella	7 (26.9%)
	• CoNS	17 (65.4%)
	• E. coli	1 (3.8%)
	• Acinetobacter	1 (3.8%)

*CoNS* Coagulase-negative staphylococci

Serum vitamin D level was statistically lower in septic neonates with respiratory distress. Newborns with poor feeding, respiratory difficulties, lethargy, cardiovascular symptoms of sepsis **(**tachycardia or bradycardia & hypotension), and mottling had significantly lower vitamin D levels than those without (*p* < 0.05). Even neonates with positive blood cultures had significantly lower vitamin D levels than newborns with negative blood cultures (*p* = 0.001) (Table 4).

**Table 4:** The association between serum vitamin D level & clinical findings & blood culture among sepsis and control groups (*N* = 90)

Items	Serum vitamin D levels in sepsis & control groups *(ng/ml)*	Test of significance
	*Median (min–max)*	
**Primary clinical manifestation**		
• **Respiratory distress**	15.3 (6.1 – 49.6)	
• **Jaundice**	30.1 (5.1 – 107)	KW = 17.0
• **Symptoms of sepsis**	12.1 (11 – 16)	***P*** = 0.001*
• **Transient tachypnea of the newborn**	28.3 (13.5 – 57.2)	
**Poor feeding**		
• **No**	30.2 (5.1 – 107)	Z = 3.5
• **Yes**	16.8 (6.1 – 57.2)	***P*** = 0.001*
**Respiratory difficulties**		
• **No**	25.8 (5.1 – 107)	Z = 2.3
• **Yes**	19.1 (6.1 – 57.2)	***P*** = 0.02*
**Jaundice**		
• **No**	14.2 (6.1 – 57.2)	Z = 4.3
• **Yes**	30.2 (5.1 – 107)	***P*** ≤ 0.001*
**Lethargy**		
• **No**	29.5 (7.5 – 107)	Z = 3.6
• **Yes**	15.7 (5.1 – 49.6)	***P*** ≤ 0.001*
**Cardiovascular symptoms of sepsis** **(**tachycardia or bradycardia & hypotension)		
• **No**	30.5 (7.6 – 107)	Z = 5.1
• **Yes**	14.2 (5.1 – 49.6)	***P*** ≤ 0.001*
**Mottling**		
• **No**	30.5 (5.1 – 107)	Z = 4.4
• **Yes**	14.8 (6.1 – 49.6)	***P*** ≤ 0.001*
**Blood culture**		
• **No blood culture ** ***(control group)***	30.4 (5.1 – 107)	KW = 16.7
• **Negative**	19.1 (7.5 – 57.2)	***P*** = 0.001*
• **Positive**	13.6 (6.1 – 38.1)	

^*^ Significant *p* ≤ 0.05. Z: Mann Whitney test. *KW* Kruskal–Wallis Test

There was a significant difference between deficient vitamin D status (vit D level < 20 ng/ml.), insufficient status (vit D laid between 20—30 ng/ml.), and sufficient vitamin D status (vit D > 30 ng/ml.) groups regarding duration of NICU stay: it was longer in neonates with deficient vitamin D status (12 days vs 6.5 days in neonates with sufficient vitamin D status). Also, CRP was significantly higher in newborns with deficient vitamin D status, with a median value of 42 g/dl compared to 12 g/dl in newborns with sufficient vitamin D status (Table 5).

**Table 5:** Association between serum vitamin D status & clinical and laboratory parameters among sepsis & control groups (*N* = 90)

Parameters	Serum Vitamin D status in sepsis and control groups			Test of significance
	**Deficient** (< *20 ng/ml.)*	**Insufficient** *(20—30 ng/ml.)*	**Sufficient** *(*> *30 ng/ml.)*	
**Gestational age ** ***(weeks). Mean*** ** ± ** ***SD***	37.5 ± 0.6	37.4 ± 0.6	37.6 ± 0.6	F = 1.0 *P* = 0.37
**Apgar score (at 1-min) M** ***ean*** ** ± ** ***SD***	8.1 ± 0.9	8.3 ± 0.8	8.4 ± 0.8	F = 0.82 *P* = 0.44
**Apgar score (at 5 min) M** ***ean*** ** ± ** ***SD***	9.5 ± 0.6	9.5 ± 0.5	9.6 ± 0.5	F = 1.1 *P* = 0.35
**Temperature ** ***mean*** ** ± ** ***SD***	37.1 ± 0.2	37 ± 0	37 ± 0	F = 1.5 *P* = 0.24
**Length of NICU stay ** ***(days) Median (min–max)***	12 (2 – 22)	9 (2 – 21)	6.5 (2 – 18)	KW = 12.7 ***P*** = 0.002*
**CRP level in the sepsis group ** ***(g/dl)***				KW = 8.9
***Median (min–max)***	42 (6 – 96)	24 (12 –48)	12 (6 – 48)	***P*** = 0.01*

^*^Significant *p* ≤ 0.05. F: One-way ANOVA test. *KW* Kruskal–Wallis Test, *NICU* Neonatal intensive care unit

**Fig. 1:**
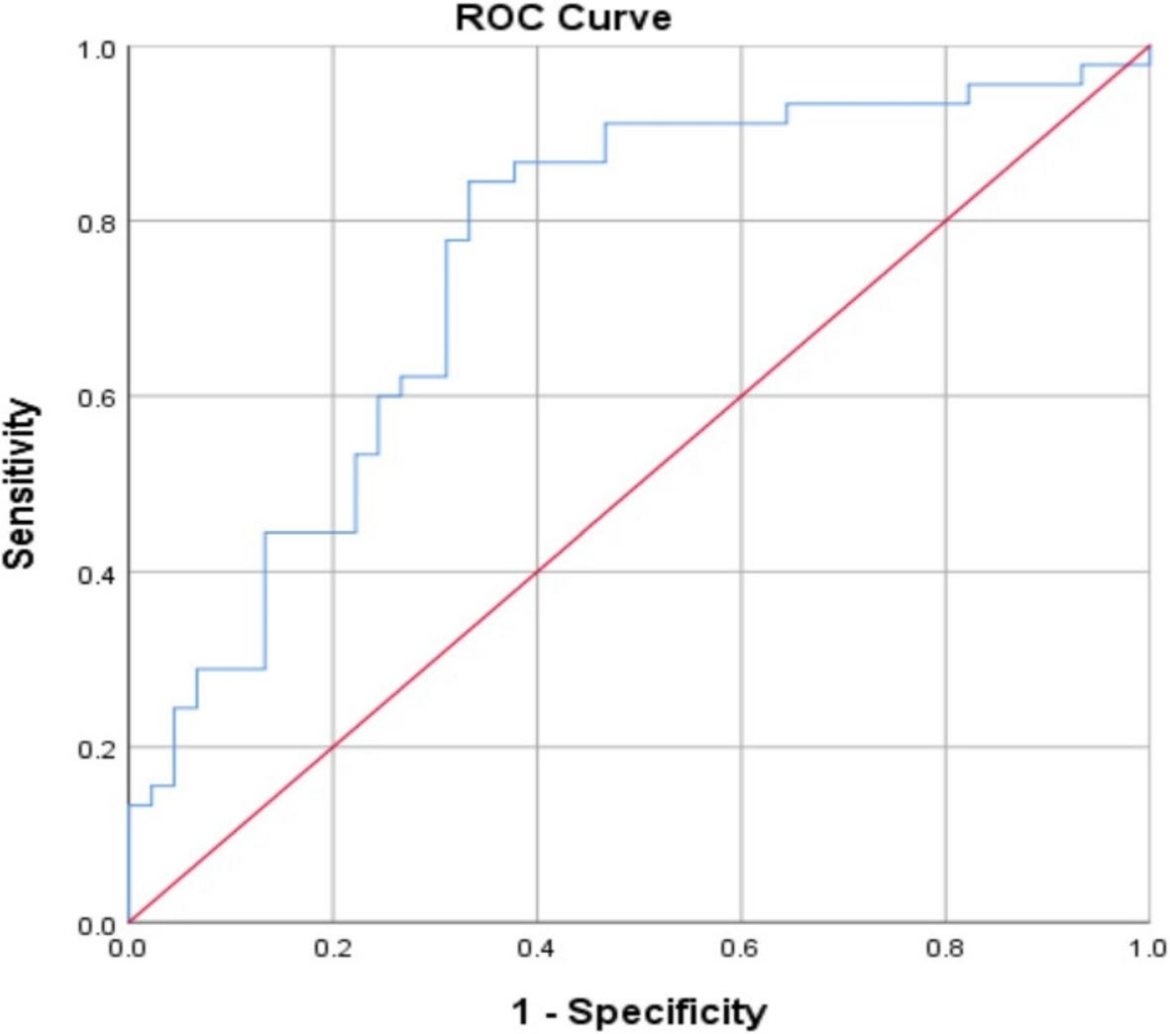
Receiver operating characteristic (ROC) curve for prediction of neonatal sepsis using serum vitamin D levels in the studied groups

The AUC for serum vitamin D in the prediction of neonatal sepsis was 0.76 at a cutoff < 19.7(ng/ml). (Table 6 and Fig. 1).

**Table 6:** Receiver operating characteristic (ROC) curve for prediction of neonatal sepsis using serum vitamin D levels in the studied groups (*N* = 90)

	**AUC**	**95% CI**		**Cutoff**	**Sensitivity**	**Specificity**	**PPV**	**NPV**	**Accuracy**
		**Lower**	**Upper**						
**Serum** **Vitamin D level** *(ng/ml)*	0.76	0.65	0.86	< 19.7	84.4%	66.7%	81.1	71.7	75.6%

*AUC* area under the curve, *CI* confidence interval, *PPV* positive predictive value, *NPV* negative predictive value

The independent predictors of sepsis among the studied cases were serum Vitamin D level (AOR = 2.8), vitamin D status (Deficient < 20 ng/ml) (AOR = 4.5), presence of respiratory difficulties (AOR = 2.1), and mottling (AOR = 1.9). The overall percent prediction of sepsis by them combined was 75.6% (Table 7).

**Table 7:** Binary logistic regression in the prediction of risk factors of sepsis among the studied group

Items	Β	*P*	AOR (95% CI)
**Apgar score at 1 min**	- 0.7	0.99	0.18 (0.01 – 1.2)
**Length of NICU stay ** ***(days)***	- 3.5	0.98	0.11 (0.01 – 1.3)
**Serum Vitamin D level ** ***(ng/ml)***	1.7	0.001*	2.8 (1.13 – 3.6)
**Vitamin D status**			
**• Deficient level (< 20** *** ng/ml*** **)**	- 2.3	≤ 0.001*	4.5 (3.03 – 5.9)
**• Insufficient level (20 – 30** *** ng/ml*** **)**	0.15	0.81	1.2 (0.33 – 4.1) 1
**• Sufficient level (> 30** *** ng/ml*** **) (r)**			
**Poor feeding**			
**• No (r)**			1
**• Yes**	0.45	0.74	1.6 (0.11 – 22.2)
**Respiratory difficulties**			
**• No (r)**			1
**• Yes**	- 2.5	0.04*	2.1 (1.5 – 2.91)
**Jaundice**			
**• No (r)**			1
**• Yes**	1.7	0.25	1.6 (0.30 – 102.3)
**Lethargy**			
**• No (r)**			1
**• Yes**	1.8	0.30	1.3 (0.20 – 166.6)
**Cardiovascular symptoms of sepsis** **(**tachycardia or bradycardia & hypotension)			
**• No (r)**			1
**• Yes**	- 1.4	0.07	0.09 (0.01 – 1.2)
**Mottling**			
**• No (r)**			1
**• Yes**	- 1.9	0.01*	1.9 (1.5 – 3.32)
**Model** χ^**2**^ ** = 71.6 Total** ***p*** **≤ 0.001**			
**Constant = 2.01**			
**Overall predicted percentage = 75.6%**			

*r* reference group, *CI* Confidence interval, *AOR* Adjusted Odds ratio

## Discussion

Vitamin D is essential to the optimum function of the immune system, especially the innate immune system which performs efficiently by stimulating the production of antimicrobial peptides in epithelial cells, neutrophils, and macrophages. Hence, vitamin D insufficiency may be considered a substantial risk factor for acquiring infections [16].

This study aimed to correlate the different levels of serum vitamin D with the development of EOS in full-term AGA newborns. In our research, there was no significant difference in the mode of delivery or sex between the cases and controls. This was in line with the findings of Kumar et al. [17]. The current study found newborns with sepsis having a statistically significant decrease in first-minute Apgar scores than controls. Similarly, other studies found significant associations between lower Apgar scores at the first and fifth minutes and the development of neonatal sepsis [18–20]. The innate immune system which is the first line of defense is immature at birth. In addition, the adaptive immune system is still not able to efficiently respond to T-cell-dependent antigens. In addition, the WHO classified low Apgar scores as an indicator of perinatal asphyxia**.** Thus, newborns with low Apgar scores may be at greater risk of infection [21–24].

Chen et al. reported that vitamin D administration during pregnancy led to a considerable decline in the amount of circulating maternal serum CRP,. This fact reveals the association between vitamin D deficiency and inflammation, infection, and septic shock [25]. The current study identified an association between low vitamin D levels and high neonatal serum CRP, in line with previous studies that found an inverse relationship between CRP levels in septic neonates and blood vitamin D levels [17, 26, 27]. While, Grzanka et al., concluded that there was no significant relationship between vitamin D concentrations and CRP and attributed this finding to the small sample size and single evaluation of vitamin D concentration [28].

In the current research, serum vitamin D levels in the sepsis group were considerably lower than in the control one. This result was consistent with other researches which concluded that full-term newborns with low serum vitamin D levels were more prone to the risk of infection [5, 17, 29, 30]. According to Soliman et al., a multivariate logistic regression analysis used to predict neonatal sepsis showed that a decline in serum vitamin D levels was substantially related to the risk of sepsis in newborns [26]. Another study explained this association by the disruption of the macrophage function and the inflammatory cytokines generation that can result from a vitamin D deficit [7]***.***

We observed in the EOS newborns different statuses of vitamin D: 66% were in the vitamin D deficient range, 11% in the insufficient one, and 22.2% in the sufficient subgroup. Vitamin D insufficiency elevated the risk of EOS by 1.44 times, while vitamin D deficiency elevated the risk of EOS to 53.44 times. As well, Kumar et al. reported the mean serum vitamin D levels were considerably lower in neonates with EOS compared to healthy controls. Out of 100 neonates with EOS, 77.0% had vitamin D deficiency, 23.0% insufficiency, and none had appropriate levels, while in the one hundred healthy controls vitamin D levels were adequate in 31%, insufficient in 28%, and deficient in 41.0% [17].

The link between serum vitamin D levels and EOS in newborns without maternal risk factors has been furthermore investigated by Sarwade et al. They concluded that in preterm and full-term ill neonates, without maternal risk factors, severe vitamin D deficiency of less than 10 ng/ml was considerably correlated with culture-positive EOS in neonates in the first three days of life [31].

In this study, there was an inverse correlation between positive blood culture results and vitamin D levels. This was in agreement with Moromizato et al., who observed a higher rate of infection and culture positivity among vitamin D-deficient patients [32]. Also, mean vitamin D levels were lower among neonates who suffered septic shock confirmed by culture than those with negative cultures [16].

The results of blood cultures in this study reported that 57.8% of those within the sepsis group had positive blood cultures. These results are higher than those reported by Betty and Inderpreet who found that culture-proven sepsis occurred in only 21% of cases with sepsis [33]. The sensitivity of blood cultures in neonatal sepsis is low and depends on the timing and number of cultures taken, blood volume, technique, temperature, culture medium, and organism density. Furthermore, the implementation of peripartum maternal antimicrobial practice makes the diagnostic value of neonatal blood cultures unreliable [1]. In the current study, out of 26 patients with positive blood cultures, 65.4% and 26.9% had coagulase-negative staphylococci (CoNS) and klebsiella respectively. Lee et al. also reported that Gram-positive organisms were the most predominant organisms of EOS in Korea. CoNS was noted as the highest, followed by Staphylococcus aureus [34].

An inverse correlation between serum vitamin D levels and the duration of NICU stay was reported in the current study. In contrast, Saadat et al. found no correlation between vitamin D levels and the length of hospital stay [35].

This study concluded that a serum vitamin D level of 19.7 ng/mL had an AUC of 0.76 for the prediction of EOS with 84.4% sensitivity and 66.7% specificity. The cutoff value of vitamin D as a measure of the risk of developing neonatal sepsis was reported by Behera et al. to be 15.48 ng/ml, with 80% sensitivity and 99.9% specificity [36]. Additionally, Soliman et al. observed that vitamin D, at a cutoff point of 18.75 ng/ml, had a 100% sensitivity, and 80% specificity for the diagnosis of newborn sepsis [26]. Another case–control study which recruited sixty-two infants with EOS and the same number as a control group, revealed that vitamin D levels were considerably lower in EOS with a cutoff value of 25 ng/ml, 88.7% sensitivity, and 79% specificity [4].

In this study, the independent predictors of sepsis among the studied group were serum vitamin D level (AOR = 2.8), vitamin D deficient category (< 20 ng/ml) (AOR = 4.5), presence of respiratory difficulties (AOR = 2.1), and mottling (AOR = 1.9). The overall value of predicted sepsis by these factors combined was 75.6%. Also, it concluded that vitamin D deficiencies and insufficiencies considerably raise the risk of EOS.

Finally, there are few limitations to this study, the first of which is the absence of measurements of maternal serum vitamin D levels. Second, the sample size is small. Lastly, the lack of serum vitamin D level measurement after vitamin D administration to the septic neonates and assessing its correlation with clinical and laboratory parameters of neonatal sepsis hindered revealing its effect on immune modulation.

## Conclusion

According to this study, full-term newborns with EOS had considerably lower vitamin D levels than healthy controls. However, this cannot be considered a diagnostic parameter (the diagnosis is still challenging for neonatologists) but a valid support to identify the subjects at major risk. Furthermore, vitamin D maternal supplementation, along with effective preventive measures (ie breastfeeding, intrapartum antibiotic prophylaxis for group B-streptococcal infection, etc.) may lower the risk of neonatal infections and improve morbidity and mortality rates [6, 7, 37].

## Data Availability

Data is available upon request.

## Abbreviations

AOR: Adjusted odds ratios
AUC: Area under the curve
CBC: Complete blood count
EOS: Early-onset sepsis
NICUs: Neonatal Intensive Care Units
ROC: Receiver operating characteristic
SD: Standard deviations

## References

[1] Celik IH, Hanna M, Canpolat FE, Pammi M (2022). Diagnosis of neonatal sepsis: the past, present and future. Pediatr Res..

[2] Milton R, Gillespie D, Dyer C, Taiyari K, Carvalho MJ, Thomson K (2022). Neonatal sepsis and mortality in low-income and middle-income countries from a facility-based birth cohort: an international multisite prospective observational study. Lancet Glob Heal.

[3] Serra G, Miceli V, Albano S, Corsello G (2019). Perinatal and newborn care in a two years retrospective study in a first level peripheral hospital in Sicily (Italy). Ital J Pediatr.

[4] Jeengar B, Gothwal S, Meena KK, Garg VK, Athwani V, Gupta ML (2021). Vitamin D levels and early onset sepsis in newborns. J Neonatol.

[5] Cizmeci MN, Kanburoglu MK, Akelma AZ, Ayyildiz A, Kutukoglu I, Malli DD (2015). Cord-blood 25-hydroxyvitamin D levels and risk of early-onset neonatal sepsis: a case–control study from a tertiary care center in Turkey. Eur J Pediatr.

[6] Myint AA (2018). Serum vitamin D levels in term neonates with early onset sepsis. Pediatr Neonatal Biol Open Access.

[7] Cetinkaya M, Cekmez F, Buyukkale G, Erener-Ercan T, Demir F, Tunc T (2015). Lower vitamin D levels are associated with increased risk of early-onset neonatal sepsis in term infants. J Perinatol.

[8] Seliem MS, Abdel Haie OM, Mansour AI, Salama SSME (2016). The relation between vitamin D level and increased risk for early-onset neonatal sepsis in full-term infants. Med Res J.

[9] Tayel SI, Soliman SE, Elsayed HM (2018). Vitamin D deficiency and vitamin D receptor variants in mothers and their neonates are risk factors for neonatal sepsis. Steroids.

[10] Lee SY, Kim HE, An SH (2018). The association between vitamin D levels and neonatal early-onset sepsis: a systematic review and meta-analysis. Korean J Clin Pharm.

[11] Gamal TS, Madiha AAS, Hanan MK, Abdel-Azeem MEM, Marian GS (2017). Neonatal and maternal 25-oh vitamin d serum levels in neonates with early-onset sepsis. Children.

[12] Gitto E, Karbownik M, Reiter RJ, Xian Tan D, Cuzzocrea S, Chiurazzi P (2001). Effects of melatonin treatment in septic newborns. Pediatr Res.

[13] Serra G, Memo L, Antona V, Corsello G, Favero V, Lago P (2021). Jacobsen syndrome and neonatal bleeding: report on two unrelated patients. Ital J Pediatr.

[14] Piro E, Serra G, Schierz IAM, Giuffrè M, Corsello G (2019). Fetal growth restriction: A growth pattern with fetal, neonatal and long-term consequences. EuroMediterranean Biomed J.

[15] Odabasi IO, Bulbul A (2020). Review neonatal sepsis. Sisli Etfal Hast Tip Bul.

[16] Gupta DVK, Dhaneria DM (2020). Study of serum vitamin d levels and its association with neonatal sepsis among newborns. Pediatr Rev Int J Pediatr Res..

[17] Kumar A, Narang GS, Singh G, Virk N (2019). Association of vitamin D deficiency with early onset sepsis in term neonates. Int J Contemp Pediatr.

[18] Adatara P, Afaya A, Salia SM, Afaya RA, Konlan KD, Agyabeng-Fandoh E (2019). Risk factors associated with neonatal sepsis: a case study at a specialist Hospital in Ghana. Sci World J.

[19] Yismaw AE, Abebil TY, Biweta MA, Araya BM (2019). Proportion of neonatal sepsis and determinant factors among neonates admitted in University of Gondar comprehensive specialized hospital neonatal Intensive care unit Northwest Ethiopia 2017. BMC Res Notes.

[20] Rafi MA, Miah MMZ, Wadood MA, Hossain MG (2020). Risk factors and etiology of neonatal sepsis after hospital delivery: A case-control study in a tertiary care hospital of Rajshahi, Bangladesh. PLoS One..

[21] Mamo SA, Teshome GS, Tesfaye T, Goshu AT (2022). Perinatal asphyxia and associated factors among neonates admitted to a specialized public hospital in South Central Ethiopia: A retrospective cross-sectional study. PLoS One..

[22] Gutbir Y, Wainstock T, Sheiner E, Segal I, Sergienko R, Landau D (2020). Low Apgar score in term newborns and long-term infectious morbidity: a population-based cohort study with up to 18 years of follow-up. Eur J Pediatr.

[23] Mitra DK, Mullany LC, Harrison M, Mannan I, Shah R, Begum N (2018). Incidence and risk factors of neonatal infections in a rural Bangladeshi population: a community-based prospective study. J Heal Popul Nutr.

[24] Mustefa A, Abera A, Aseffa A, Abathun T, Degefa N, Tadesse H (2020). Prevalence of neonatal sepsis and associated factors amongst neonates admitted in arbaminch general hospital, arbaminch, southern Ethiopia, 2019. J Pediatr Neonatal Care.

[25] Chen N, Wan Z, Han SF, Li BY, Zhang ZL, Qin LQ (2014). Effect of vitamin D supplementation on the level of circulating high-sensitivity C-reactive protein: a meta-analysis of randomized controlled trials. Nutrients..

[26] Soliman Y, Sakr M, Emran T, El Samanoudy M (2019). Impact of serum level of vitamin D on term neonates with early onset sepsis. Int J Med Arts..

[27] Tao RX, Zhou QF, Xu ZW, Hao JH, Huang K, Mou Z (2015). Inverse correlation between vitamin D and C-reactive protein in newborns. Nutrients.

[28] Grzanka A, Machura E, Mazur B, Misiolek M, Jochem J, Kasperski J (2014). Relationship between vitamin D status and the inflammatory state in patients with chronic spontaneous urticaria. J Inflamm (Lond).

[29] Yang LR, Li H, Yang TY, Zhang T, Zhao RC (2016). Relationship between Vitamin D deficiency and early-onset neonatal sepsis. Chinese J Contemp Pediatr.

[30] Upala S, Sanguankeo A, Permpalung N (2015). Significant association between vitamin D deficiency and sepsis: a systematic review and meta-analysis. BMC Anesthesiol.

[31] Sarwade A, Gosai M, Gohil J (2019). Vitamin D levels in early onset neonatal sepsis without maternal risk factors: a case-control study. Vitam Miner.

[32] Moromizato T, Litonjua AA, Braun AB, Gibbons FK, Giovannucci E, Christopher KB (2014). Association of low serum 25-hydroxyvitamin D levels and sepsis in the critically ill. Crit Care Med.

[33] Chacko B, Sohi I (2005). Early onset neonatal sepsis. Indian J Pediatr.

[34] Lee SM, Chang M, Kim KS (2015). Blood culture proven early onset sepsis and late onset sepsis in very-low-birth-weight infants in Korea. J Korean Med Sci..

[35] Saadat H, Mehrvari T, Goodarzi R, Kheiry F (2021). The comparison of serum vitamin D level in the term neonates with and without sepsis in children hospital of Bandar Abbas city, Iran from 2016 to 2017. Hormozgan Med J.

[36] Behera CK, Sahoo JP, Patra SD, Jena PK (2020). Is lower vitamin D level associated with increased risk of neonatal sepsis? A prospective cohort study. Indian J Pediatr.

[37] Aly H, Abdel-Hady H (2015). Vitamin D and the neonate: an update. J Clin Neonatol.

